# Mortality in long-distance running races in Sweden - 2007–2016

**DOI:** 10.1371/journal.pone.0195626

**Published:** 2018-04-09

**Authors:** Finn Nilson, Mats Börjesson

**Affiliations:** 1 Department of Environmental and Life Sciences, Karlstad University, Karlstad, Sweden; 2 Department of Neuroscience and Physiology, Sahlgrenska Academy, University of Gothenburg, Gothenburg, Sweden; 3 Department of Food, Nutrition and Sport Science, Sahlgrenska University Hospital, Gothenburg, Sweden; University of Tampere, FINLAND

## Abstract

**Background:**

During the last decade, an increasing popularity of marathons has been seen. Although running has been shown to have considerable positive health effects, the risk of sudden death, most often due to sudden cardiac arrests, is also a risk runners expose themselves to. Whilst there are some studies on the mortality amongst long-distance runners, much of the evidence is dated. Given the increased popularity in running during the 21^st^ century as well as the improvements in medical care at marathons, more knowledge is required on the mortality risk.

**Materials and method:**

Publicly available racing and news databases were used to identify the number of entrants and finishers in half to full marathons in Sweden between 2007 and 2016 and the number of deaths that occurred in conjunction with the races.

**Results:**

A total of 1,156,271 runners entered a long distance (21-42km) running race in Sweden between 2007 and 2016, and 834,412 runners finished the races (72.2%). A large majority of the finishers (677,050 (81%)) competed in distances under a full marathon. Two deaths occurred during the time period, meaning that the death rate was 0.24 (95% confidence interval 0.04–0.79) per 100,000 finishers.

**Conclusions:**

This study can show that death rates in long distance running races between 2007 and 2016 in Sweden are very low, compared to previous studies. When added to the existing literature, the combined picture suggests a general downward trend in the risk of death during marathons since the 1980s.

## Background

During the last decade, an increasing global popularity of endurance events such as cycling, Nordic skiing and running half, full and ultra-marathons, has been seen [1], with a particular increase in the number of both half and full city marathons [2]. Endurance exercise, and running especially, has been shown to have considerable positive effects on individuals with regards to general mortality [3] as well as general physical and mental health [4], but also specifically reduce the risk of hypertension, hyperlipidemia and diabetes [5]. However, although events that promote physical activity, such as marathons, are important, particularly from a public health perspective [6, 7], due to the nature of competition and the prolonged physical exertion, medical encounters are inevitable during endurance events [8].

According to two reviews of the literature, the risk of medical encounters at endurance events increases with the length of the race. Whilst half-marathons (21km) and Nordic skiing races (55km) have a risk of between 1 and 5%, marathons (42km) have a risk of between 1 and 20% [9–11]. Of these medical encounters at marathons, the overwhelming majority are moderate and musculoskeletal [12]. However less common, the risk of sudden death, most often due to cardiac events, is also existent. These deaths often result in considerable media attention and may thereby affect the general attitudes towards running therefore consequently reducing the positive public health effects.

The risk of death at marathons and half-marathons has been studied in a number of studies [1, 13–18] and a review article has also been published on the subject [19]. Based on these studies, the risk of sudden cardiac death at marathons ranged between 0.6 and 1.9 per 100,000 runners. However, the reported figures in these papers are all based on data from the USA or the UK. Also, three of the studies include data from as far back as the 1970’s [13, 14, 16] and two include data from the 1980’s [17, 18]. Running, and especially long distance running, has increased in popularity considerably during the 21^st^ century and concerns have been raised that many of the newer marathon runners are less physically fit and less well trained therefore perhaps increasing the risk of sudden cardiac events [1]. However, simultaneously, the survival rates of cardiac arrests during both running races and during training has increased considerably during the last 15 years, largely due to the availability of defibrillators and knowledge regarding CPR from bystanders [20, 21]. With this in consideration, it may be more correct to isolate the studies merely including data from the 2000’s. These two studies [1, 15] report an incidence of 0.58 and 0.75, i.e. a considerably lower rate than articles including older data.

Whilst indications exist of a reduction in death rates during marathons, there are also indications of higher rates of cardiac arrests at half-marathons compared to marathons [11, 20]. Shirakawa et al. [20] suggest that this could be due to a higher percentage of untrained runners competing in half marathons. However, these results contradict other studies that show the risk of cardiac arrests to be significantly higher at marathons compared to half-marathons [15, 22]. Therefore, given that some discrepancy seems to exist in the differing risks between different types of races, and the fact that mortality rates at long distance running races seem to have decreased, this study aims to investigate mortality rates at running races between 21 and 42km in Sweden between 2007 and 2016.

## Materials and method

The dataset for this study was based on data from two datasets and the methodology was largely based on the methodology used by Mathews et al [1], i.e. systematically searching media reports for deaths during running races. Although using media reports has previously been criticised as unreliable [23], with regards to assessing sudden deaths at running races during the studied time period in Sweden, several aspects indicate that the chosen method is reliable. Firstly, when using a similar method, Mathews et al [1] found results that were on par with results using other data collection methods [13, 15, 16]. Secondly, the chosen study period only covers a time period in which comprehensive, unchanged internet media coverage has existed in Sweden, therefore reducing the risk of under-coverage due to technological advancements [23].

In order to identify deaths having occurred during a race rather than during exercise or training, all official running races with at least 1000 entrants were identified using the Swedish Athletic Associations official lists from 2007 to 2016. These races were then categorized according to length into full marathons (42km) or half marathons to full marathons (21-42km). Races over shorter distances than 21km were omitted from the analyses. The number of entrants per year for each race was collected from the Swedish Athletic Association and the number of participants and finishers were collected from either the Swedish Athletic Association, the race website or media reports.

In order to identify the number of deaths during running races in Sweden, the Retriever Research database was used. Retriever Research is a media database including all online and printed articles from Swedish and Scandinavian newspapers. The database was searched by using the race name and (in Swedish) the terms of “die*”, “dea*” or “fatal*”. The results were then manually checked for each year for reported deaths of runners during the race. In similarity to Mathews et al, deaths were only included if these occurred within 24 hours of the event (according to Mathews et al, in their data, one case was omitted due to this criteria) [1]. Also in similarity to Mathews et al [1], deaths confirmed only by a single news source were excluded. For this study, this meant that one potential case was excluded. This particular case was a death that was reported several years after the supposed event. Despite actively searching databases for reports on this death, no other reports of the death were found.

From the media reports, information on runners’ deaths including the name, date, location of the race in which the death occurred, age, gender, and cause of death of the runner was collected. From the datasets, descriptive statistics were calculated and presented, comparing different years as well as different race lengths. All analyses were performed in SPSS version 22.

## Results

### Number of competitors in long distance races

**Fig 1:**
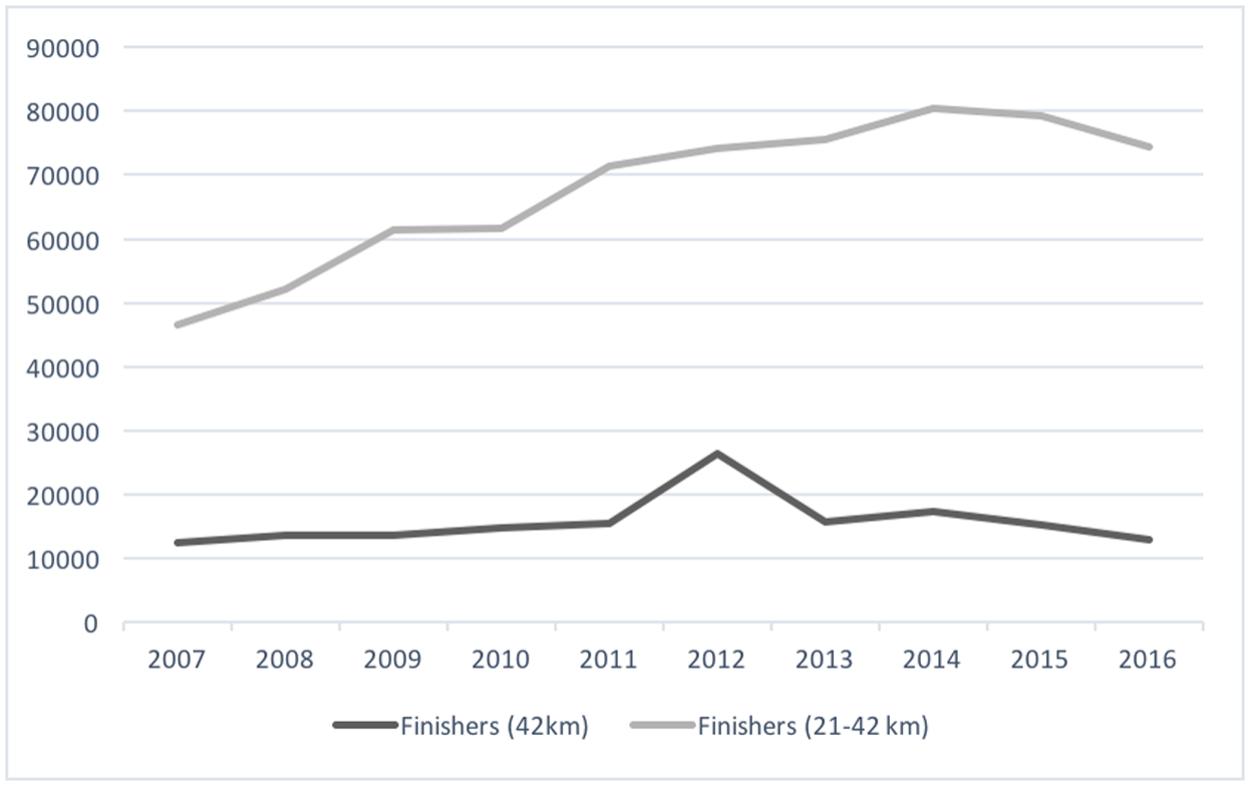
Number of finishers of full marathons and half—Full marathons in Sweden between 2007 and 2016.

Between 2007 and 2016 a total of 1,156,271 runners entered a long distance (21-42km) running race in which at least 1000 people finished, and 834,412 runners finished the races (72.2%). A large majority of the finishers (677,050 (81%)) competed in distances under a full marathon (all distances under a full marathon were half marathons except one race that was 30km). As can be seen in Fig 1, the number of finishers of 21-42km races increased by 72.5% between 2007 and 2014, to thereafter level off. In terms of finishers of 42km races, 2012 stands out, the cause of which was a one-off race to celebrate the centenary of the 1912 Stockholm Olympics. Disregarding that race, the number of finishers increased by 39.5% between 2007 and 2014.

**Fig 2:**
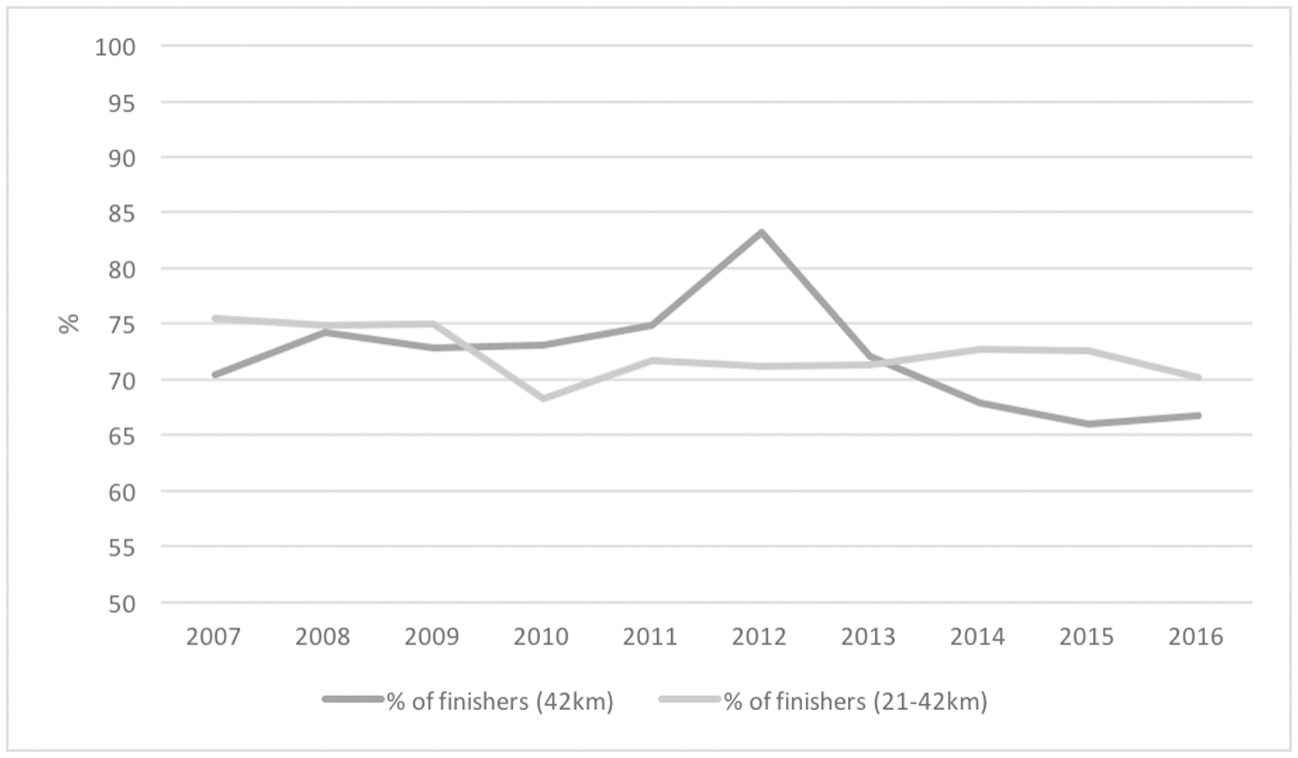
Percentage of entrants to marathons and half—Full marathons who finish the race.

In terms of the percentage of finishers in relation to the number of entrants, as can be seen in Fig 2, this has decreased during the studied time period.

### Race-related deaths

During the period 2007–2016, a total of two deaths occurred during long distance (21-42km) running races in Sweden (Table 1). Both of the victims died of cardiac events and both were men. One of the victims died after the finish line and the other halfway along the course. According to the media reports, both received qualified medical attention rapidly after collapsing.

**Table 1:** Deaths during (or within 24 hours after) long distance (21-42km) running races in Sweden 2007–2016.

Year	Race type	Sex	Age	Location of collapse
2016	21-42km	M	50	After completing 50% of the race
2014	21-42km	M	27	Post finish line

Given that no full marathon runners died, incidence rates are obviously not possible to calculate for the full marathon population. However, for the 21-42km population, as well as the overall death rate for the 10-year period, rates were possible to calculate (Table 2). Overall, the death rate for all races between half and full marathons the 10-year period was 0.24 (95% confidence interval 0.04–0.79) per 100,000 finishers.

**Table 2:** Death rates and 95% confidence intervals at long distance (21-42km) running races in Sweden 2007–2016.

	No of deaths	No of runners	Incidence rates, /100,000 (95% CI)	Expected no of deaths	Exp. no. of deaths (95% CI) based on the results of Kim et al	Exp. no. of deaths (95% CI) based on the results of Matthews et al
21-42km (entrants)	2	939,412	0.21 (0.04–0.70)	1.97 (0.34–6.61)	2.58 (1.60–3.95)	n/a
21-42km (finishers)	2	677,050	0.30 (0.05–0.98)	2.03 (0.34–6.64)	n/a	n/a
42km (entrants)	0	216,859	n/a	n/a	1.26 (0.82–1.86)	n/a
42km (finishers)	0	157,362	n/a	n/a	n/a	1.18 (0.80–1.69)
All (entrants)	2	1,156,271	0.17 (0.03–0.57)	1.97 (0.35–6.59)	4.47 (3.26–5.98)	n/a
All (finishers)	2	834,412	0.24 (0.04–0.79)	2.00 (0.33–6.59)	n/a	n/a

Based on the incidence rates and the 95% confidence intervals, the expected number of deaths were calculated in the different categories. The different categories were chosen in order to be comparable to the two previous studies given that Kim et al used entrant data and Matthews et al used finisher data [1, 15]. Also, whilst Kim et al also studied half marathons, Matthews et al merely included full marathons [1, 15]. As can be seen in Table 2, although the number of deaths were lower in our dataset compared to the expected results based on Kim et al and Mathews et al, given the large confidence intervals, the results are not significantly lower than the two comparable studies. Also noteworthy, even if no reported deaths have occurred in full marathons in Sweden during the studied time period, this cannot be determined to be significantly lower than the rates that previous studies have reported.

## Discussion

**Fig 3:**
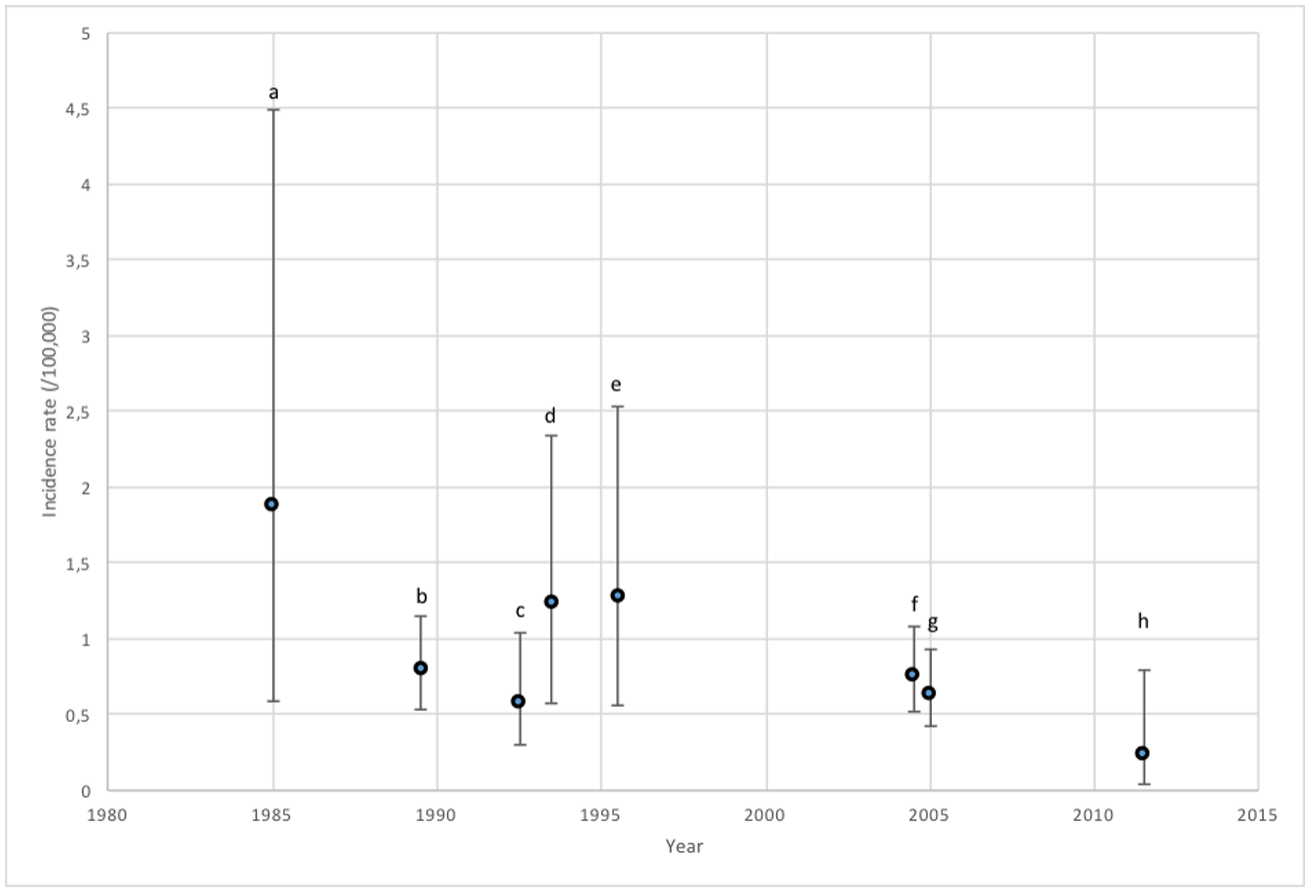
Recalculated mortality incidence rates (based on finishers) and 95% confidence intervals from previously published articles *(a*: *Maron et al*. *(1996)* [14], *b*: *Redelmeier & Greenwald (2007)* [16], *c*: *Webner et al*. *(2012)* [13], *d*: *Pedoe (2007)* [17], *e*: *Roberts et al*. *(2012)* [18], *f*: *Mathews et al*. *(2012)* [1], *g*: *Kim et al*. *(2012)* [15], *n*.*b*. *based on entrant data*, *h*: *results from this study*, *n*.*b*. *21-42km)*, distributed according to the midpoint of the study’s study period.

As has been shown in this paper, deaths during long distance races in Sweden are rare. When adding the results from this study to previous studies, the evidence would suggest that death rates are considerably lower now compared to previous decades. In Fig 3, recalculated death rates and 95% confidence intervals of seven previously published articles on long distance race deaths are presented in relation to the midpoint of the years studied. Although no statistically significant differences can be seen, primarily due to the low rates, compared to previous studies the results indicate that the death rates at long distance races between 2007 and 2016 in Sweden are amongst the lowest ever published.

When comparing the results in this study to previous studies, it is important to note differences in the studied time period, the methodology but also the studied races. The previous studies on deaths during long distance running races have either been based on data from specific races such as London Marathon [17] or Twin City Marathon/Marine Corps Marathon [14, 18], alternatively by collecting information from many smaller races [1, 13, 15, 16]. As can be seen from Fig 3, the three papers studying specific (larger) races (a, d, e) have higher rates compared to the others. This would seem to be surprising given that larger, more well-known races would be expected to have a more comprehensive medical set-up. However, these races are also events that more often attract “non-runners”, i.e. individuals who have not previously run a marathon, who run for charity, etc. Therefore, it is possible that less well-trained athletes are competing in the larger races and thereby increase the rates of sudden cardiac arrests (SCA). This is supported by comparing data from Roberts et al [18] and Webner et al [13]. Despite reporting data from roughly the same time period, Roberts et al report an incidence rate of SCA of 2.6 per 100,000 whilst Webner et al report a SCA rate of 1.75 per 100,000 [13, 18]. In terms of the data included in this paper, although all races in Sweden with over 1000 finishers were included, the number of races included per year only varied between 4 and 8. The reason for this is that few long-distance races exist in Sweden and a large majority of the competitors were from three races (Gothenburg Half-Marathon, Lidingöloppet (30km) and Stockholm Marathon). Therefore, the results from this study could be seen as both including specific races and smaller races. Unfortunately, due to the chosen methodology, it was not possible to assess the number of SCAs in the studied races.

The low incidence rates reported in this study are also considerably lower than previously reported rates of exercise-related out-of-hospital sudden cardiac deaths (SCD) where generally, rates between 1–3 per 100,000 have been reported [24, 25]. This would indicate one of two factors. Either incidence rates of cardiac arrests are lower during long distance races compared to non-race exercise, or survival rates are higher. Given the method of data collection used in this study, we unfortunately do not have the SCA incidence during the races nor the use of defibrillators and we therefore cannot ascertain either of these hypotheses. However, whilst a recent Swedish study on exercise related out-of-hospital sudden cardiac arrests (SCA) showed a survival rate of 54.3% in all ages [21], studies have shown an almost 70% survival rate with regards to cardiac arrests that occur during long distance races [13, 20]. Therefore, if survival rates at Swedish races are on par with these studies, this could explain the considerably lower SCD rates reported in this study compared to non-race exercise rates. Whether survival rates are higher during races due to organizational factors or medical preparedness from the race organizers is an aspect that needs to be studied further. Unpublished data from Gothenburg’s half marathon suggests that competitors in long distance races in Sweden are considerably more educated than the general population and contain a large number of medically trained individuals (personal communication with Gothenburg’s half marathon organizers, January 2018). Therefore, survival rates may be higher due to competence amongst bystanders or fellow runners rather than well-designed medical preparedness.

Although the results in this study are promising, there are some limitations that need to be addressed. Firstly, although the chosen data collection method has been used in previous studies, relying on media reports is less reliable than for example medical reports or medical registers. Currently, no such registers exist even though the Swedish Ambulance registry, now covering almost 100% of out-of-hospital cardiac arrests, has the potential to provide “true” figures in the future, as soon as data from the whole country is available. However, the Swedish newspaper/media coverage is considerably greater than in many other countries given the government subsidies that support regional and local media outlets [26]. Therefore, the chances that a death following a long-distance race would be missed by local media, is slim. An alternative source of information would be to contact race organizers similarly to Webner et al. [13] However, this method was rejected for two reasons. Firstly, given the low response rate from Webner et al (22%) [13], the method seems to have some flaws, and secondly, a number of the races included in this study were one-off races and contact details to organizers were not possible to find.

A further limitation is that in order to assess why the number of SCDs seems to be lower in this paper compared to previous reports, ideally, a full coverage of SCAs during the races would be preferable. With such information, it could be assessed whether, in similarity to previous studies [20, 27], the low rates of SCDs in this study are due to improved treatment of SCAs, through for example medical action plans [28], availability of defibrillators, etc. Again, such information may be available in the future from the Ambulance registry, therefore potentially answering questions related to survival rates, successful resuscitation, etc. Using an Ambulance registry, alternatively an in-patient or national death registry, could also be a future strategy in order to more reliably compare different races and studies, therefore ascertaining whether a downward trend in SCDs is true. However, in most in-patient and death registers, it is currently not possible to identify individuals who have partaken in a race or have been running long distances. Therefore, considerable adaptations to for example the ICD coding scheme will be necessary in order to achieve completely comparable datasets.

## Conclusions

This study can show that death rates in long distance running races between 2007 and 2016 in Sweden are very low, compared to previously published figures. Also, when added to the previous knowledge on the subject, the combined picture suggests a general downward trend in death rates during marathons since the 1980s. Whilst promising, more research is required in order to investigate the cause behind the low mortality rates and whether they represent low rates of SCA or high rates of survival.

## Supporting information

S1 File Dataset for marathon entrants and finishers as well as mortality data. (XLSX)
